# Assessment of bone quality with trabecular bone score in patients with inflammatory bowel disease

**DOI:** 10.1038/s41598-021-99669-z

**Published:** 2021-10-13

**Authors:** Iulia Soare, Anca Sirbu, Sorina Martin, Mircea Diculescu, Bogdan Mateescu, Cristian Tieranu, Simona Fica

**Affiliations:** 1 University of Medicine and Pharmacy Carol Davila, Bucharest, Eroii Sanitari 8, Bucharest, Romania; 2 Department of Endocrinology, Elias University and Emergency Hospital, Marasti nr 17, Bucharest, Romania; 3 Department of Gastroenterology, Fundeni Clinical Institute, Fundeni 258, Bucharest, Romania; 4 Department of Gastroenterology, Colentina Hospital, Stefan cel Mare 19-21, Bucharest, Romania; 5 Department of Gastroenterology, Elias University and Emergency Hospital, Marasti 17, Bucharest, Romania

**Keywords:** Endocrine system and metabolic diseases, Osteoporosis, Gastroenterology, Gastrointestinal diseases, Inflammatory bowel disease

## Abstract

Inflammatory bowel disease (IBD) patients have a significant risk of developing bone loss. The trabecular bone score (TBS) is a relatively new parameter used to provide information on bone quality. The study cohort included 81 patients with IBD and 81 healthy controls. Blood tests, dual-energy x-ray absorptiometry (DXA), including TBS, were assessed. Harvey–Bradshaw Index (HBI) for Crohn's disease (CD) and the Partial Mayo Score for ulcerative colitis (UC) were used for evaluation of clinical disease activity. Compared with the healthy controls, the IBD patients had lower lumbar spine (LS) bone mineral density (BMD) (1.06 ± 0.18 vs. 1.16 ± 0.15 g/cm^2^, *p* < 0.005), hip BMD (0.88 ± 0.13 vs. 0.97 ± 0.13 g/cm^2^, *p* < 0.005) and TBS (1.38 ± 0.1 vs. 1.43 ± 0.1, *p* < 0.005) values. The patients with stricturing CD had lower TBS (1.32 ± 0.13 vs. 1.40 ± 0.9, *p* = 0.03) and LS BMD (0.92 ± 0.19 vs. 1.07 ± 0.1, *p* = 0.01) values compared with those with non-stricturing CD. Multivariate regression model analysis identified HBI as independent factor associated with TBS. Our results support that all DXA parameters are lower in patients with IBD than in healthy patients. Moreover, TBS is a valuable tool for assessment of bone impairment in active CD.

## Introduction

Crohn's disease (CD) and ulcerative colitis (UC), which are the two main types of inflammatory bowel disease (IBD), are chronic gastrointestinal pathologies that can affect both young and old patients. Bone impairment is a common complication of these diseases to which multiple factors are linked, from chronic inflammatory status to high-dose glucocorticoid treatment. Osteoporosis and osteopenia are frequent findings, with the literature stating prevalence rates ranging from 12 to 42% and 77%, respectively^1^. Decreased bone mineral density (BMD) could lead to fragility fractures. In a recent meta-analysis, Hidalgo^2^ found that patients with IBD have a 32% increased risk of developing an osteoporotic fracture, more commonly in the vertebral area^3^.

Dual-energy X-ray absorptiometry (DXA) is the gold standard for the evaluation of bone loss, and BMD characterises bone quantity changes. The *Z* and *T* scores are used to establish the diagnosis of osteoporosis, osteopenia or low BMD according to the 2019 International Society of Clinical Densitometry official position for adults^4^. However, some studies have found an increased risk of osteoporotic fracture in patients with slightly decreased or even normal BMD^5^, suggesting that a more suitable parameter could predict the early onset of changes in bone quality. The bone microarchitecture requires invasive methods of assessment (e.g. bone biopsy) or expensive research tools [e.g. high-resolution peripheral quantitative computed tomography (HRpQCT)]. The trabecular bone score (TBS) was developed out of the need for a less complicated and non-invasive surrogate parameter^6^. This index is a greyscale textural analytical tool that measures lumbar spine (LS) DXA and BMD-independent predictors for both skeletal strength and risk of fractures. It has the advantage of not being influenced by lumbar osteoarthritis and degenerative changes in the LS, which can co-occur in CD.

Several studies showing the utility of the TBS in estimating bone changes due to glucocorticoid excess, hyperparathyroidism and type 2 diabetes mellitus have been conducted^7^. By contrast, few studies elucidating the role of the TBS in bone changes in IBD have been reported to date^8,9^. Literature data regarding the bone microarchitecture in IBD are also scarce, with one study showing that both cortical and trabecular BMDs assessed by HRpQCT are impaired, more severely so in CD patients^10^. The objectives of this study were thus to evaluate and compare both bone quality (as assessed by TBS) and quantity (as assessed by BMD) in patients with IBD and healthy controls as well as to describe the usefulness of the TBS in IBD bone impairment.

## Methods

### Study population

A cross-sectional study with 81 IBD patients (48 with CD and 33 with UC) and 81 healthy controls was conducted at the Elias Hospital Department of Endocrinology. The IBD patients (age range, 20–70 years) were diagnosed according to European Crohn and Colitis Organisation guidelines^11^ and were registered in one of the three participating gastroenterology tertiary centres. Exclusion criteria included a previous diagnosis of diseases affecting bone metabolism and pregnancy. The control group included 81 individuals without IBD or any other metabolic bone disorder who were matched by age, body mass index (BMI) and sex. All study participants provided their informed consent, the study protocol was conducted in accordance with the Declaration of Helsinki and was approved by the ethics committees of the Carol Davila University of Medicine and Pharmacy (Bucharest, Romania) and Elias Hospital.

### Clinical and paraclinical evaluation

The patients’ demographic characteristics, such as age at diagnosis, disease duration, medical history and previous surgery history, were obtained from their records. Patient workup included anthropometric measurements (height and weight), with the calculation of BMI; biological tests; and 25-hydroxy vitamin D measurement. The patients’ recent faecal calprotectin level was also documented, and their scores on disease activity indices, namely, the Harvey–Bradshaw Index (HBI) for CD and the Partial Mayo Scoring Index (PMSI) for UC, were calculated. The HBI considers general well-being, abdominal pain, frequency of liquid bowel movements, abdominal mass and complications (arthralgia, new fistula, abscess, etc.). The PMSI takes into account stool frequency, rectal bleeding and Physician’s Global Assessment score. The definition of clinical remission was based on a disease score of ≤ 4 in the HBI and that of < 1 in the PMSI. The Montreal classification was used to characterise age at diagnosis, disease localisation and behaviour in patients with CD as well as the extension of the disease in patients with UC.

The variables in CD were classified as follows: A1, age at diagnosis < 16 years; A2, age at diagnosis between 17 and 40 years; A3, age at diagnosis > 40 years; L1, ileal disease localisation; L2, colonic disease localisation; L3, ileocolonic disease localisation; L4, isolated upper gastrointestinal disease localisation; B1, non-penetrating/non-stricturing disease behaviour; B2, stricturing disease behaviour and B3, penetrating disease behaviour. Disease location was further categorised as colonic or non-colonic. For UC, E1 represented ulcerative proctitis; E2, left-sided UC and E3, extensive UC. To assess their previous exposure to high-dose glucocorticoids, we divided the patients into three groups according to the number of courses of their therapy (high-dose methylprednisolone at > 7.5 mg/day for > three months): group 1, no exposure/single course of therapy; group 2, with two to four courses of therapy and group 3, with more than four courses of therapy. None of the patients or controls received anti-osteoporotic treatment or vitamin D and calcium supplementation at the time of the evaluation.

### DXA assessment

The BMDs (in grams per square centimetre) of the LS and hip were evaluated using DEXA Prodigy^®^, GE Healthcare #212018. The TBS was analysed using DXA images of the LS (L1–L4) using TBS iNsight version 3.0.2.0 and calculated for the same region as that in the LS BMD assessment. According to the International Society of Clinical Densitometry, low BMD is defined by a *Z* score lower than − 2 SD in premenopausal women and men < 50 years or by *T* scores in others, with a *T* score between − 1 and − 2.5 SD for osteopenia and that of ≥ 2.5 SD for osteoporosis^4^. A vertebral fracture assessment was also performed, and patients suspected of having a fracture underwent LS X-ray or magnetic resonance imaging to confirm the diagnosis. Appendicular skeletal mass index (ASMI) was calculated according to the European Working Group on Sarcopenia in Older Patients and the International Working Group on Sarcopenia as the sum of the muscle mass of the arms and legs divided by square height^12,13^. This parameter was used to assess lean mass.

### Statistical analysis

Statistical analysis was performed using SPSS version 21.0 (SPSS). Descriptive data were presented as mean ± SD or median (interquartile range). Pearson and Spearman correlations were used for parametric and nonparametric variables, respectively. Non-normally distributed variables were logarithmically transformed before being included in linear regression analysis, which was used to determine the influence of different parameters on TBS. Between-group comparisons were carried out using an independent *t* test, analysis of variance (for normally distributed variables) and the Mann–Whitney *U* test (for nonparametric variables). For the analysis between the three subgroups, general linear model with Bonferroni correction for multiple factors was used. A *p* value of < 0.05 was considered statistically significant.

## Results

### Demographic and disease-specific characteristics of the IBD patients

The CD group consisted of 48 patients, of whom 24 (50%) were women, with a median age of 42 (IQR 23) years and a median BMI of 24 (IQR 7) kg/m^2^. According to the Montreal classification for CD, ten patients were graded as A1, 20 as A2 and 18 as A3. Non-colonic disease was present in 20 patients, whereas colonic disease was present in 28. A stricturing/penetrating pattern was found in 11 of these 48 patients, and previous intestinal surgery was documented in 20. UC group consisted of 33 patients, of whom 19 (57%) were women, with a median age of 44 (IQR 27) years and a median BMI of 25 (8) kg/m^2^.

The median HBI score of the CD patients was 3 (7). Twenty-eight patients were in remission, whereas 20 had clinically active CD. The median PMSI score of the UC patients was 2 (3), with 16 patients in remission and 17 having active UC. With regard to previous exposure to high-dose glucocorticoids, approximately one-third of the patients were categorised in group 1, 28 patients in group 2 and 23 patients in group 3. Disease characteristics of the patients are presented in Table 1.

**Table 1:** Characteristics of IBD patients.

Parameter	Crohn's disease (CD) (n = 48)	Ulcerative colitis (UC) (n = 33)
Gender (male/female)	24/24	14/19
Age (years) (IQR)	42 (23)	44 (27)
Disease-duration (years) (IQR)	8 (6)	10.3 (13)
Activity index HBI/partial Mayo score (IQR)	3 (7)	2 (3)
Active disease (patients)	20	17
Surgery (patients)	20	5
**Age at diagnosis**		
< 17 years 17–40 years > 40 years	10 20 18	
**Location**		
Non-colonic disease Colonic disease	20 28	
**Behaviour**		
Stricturing/penetrating	11	
**Montreal classification**		
E1-ulcerative proctitis E2-left-sided UC E3 extensive UC		3 16 14
**Glucocorticoid exposure**		
Group 1 Group 2 Group 3	16 17 15	14 11 8

Values are presented as median (IQR), number, according to the type of variable and the normality of distribution.

*BMI* body mass index.

### DXA parameters

#### Differences in DXA parameters between IBD patients and healthy controls

81 control subjects were included in the study, 42 women and 39 men, with a median age 38.5 (23) years and a median BMI of 24.9 (6.6) kg/m^2^. Compared with the controls, the IBD patients had significantly lower LS BMD (1.06 ± 0.18 vs. 1.16 ± 0.15 g/cm^2^, *p* < 0.005), hip BMD (0.88 ± 0.13 vs. 0.97 ± 0.13 g/cm^2^, *p* < 0.005) and TBS (1.38 ± 0.1 vs. 1.43 ± 0.1, *p* < 0.005) values. The differences in DXA parameters between CD and UC patients were not statistically significant. Low BMD was present in 49.3% of the IBD patients, compared with 23.4% of the healthy controls (*p* = 0.001). Fractures were observed in eight IBD patients (vertebral, *n* = 5; wrist, *n* = 2; hip, *n* = 1) and one control (*p* = 0.01). Further data are presented in Table 2.

**Table 2:** DXA parameters and low BMD in case and control group.

Parameter	IBD patients (n = 81)	CD patients (n = 48)	UC patients (n = 33)	Controls (n = 81)	CD vs. UC p-value	IBD vs. controls p-value
Age (years) (IQR)	43 (24)	42 (23)	44 (27)	38.5 (23)	0.5	0.3
Gender (male/female)	38/43	24/24	14/19	39/42	0.5	0.8
BMI kg/m^2^ (IQR)	24.5 (7.5)	24.4 (7.3)	25.5 (8)	24.9 (6.6)	0.8	0.3
LS BMD (g/cm^2^) (SD)	1.06 ± 0.18	1.04 ± 0.18	1.09 ± 0.17	1.16 ± 0.15	0.1	< 0.005
LS BMD T-scores (SD)	−1.05 ± 1.41	−1.18 ± 1.4	−0.87 ± 1.4	−0.2 ± 1.26	0.3	< 0.005
Hip BMD (g/cm^2^) (SD)	0.88 ± 0.13	0.87 ± 0.14	0.91 ± 0.1	0.97 ± 0.13	0.2	< 0.005
Hip BMD T-scores (SD)	−1.1 ± 0.9	−1.2 ± 1.07	−0.91 ± 0.8	−0.45 ± 1.02	0.1	< 0.005
Low BMD (%)	40 (49.3%)	27 (56.2%)	13 (39.3%)	19 (23.4%)	0.1	0.001
TBS (SD)	1.38 ± 0.1	1.38 ± 0.1	1.38 ± 0.1	1.43 ± 0.1	0.9	< 0.005

Values are presented as mean ± SD, percentage.

p value presented statistically significant differences p < 0.05.

*BMI* body mass index, *LS BMD* lumbar spine bone mineral density, *BMD* bone mineral density, *TBS* trabecular bone score.

#### Association between DXA parameters and exposure to high-dose glucocorticoids

**Figure 1:**
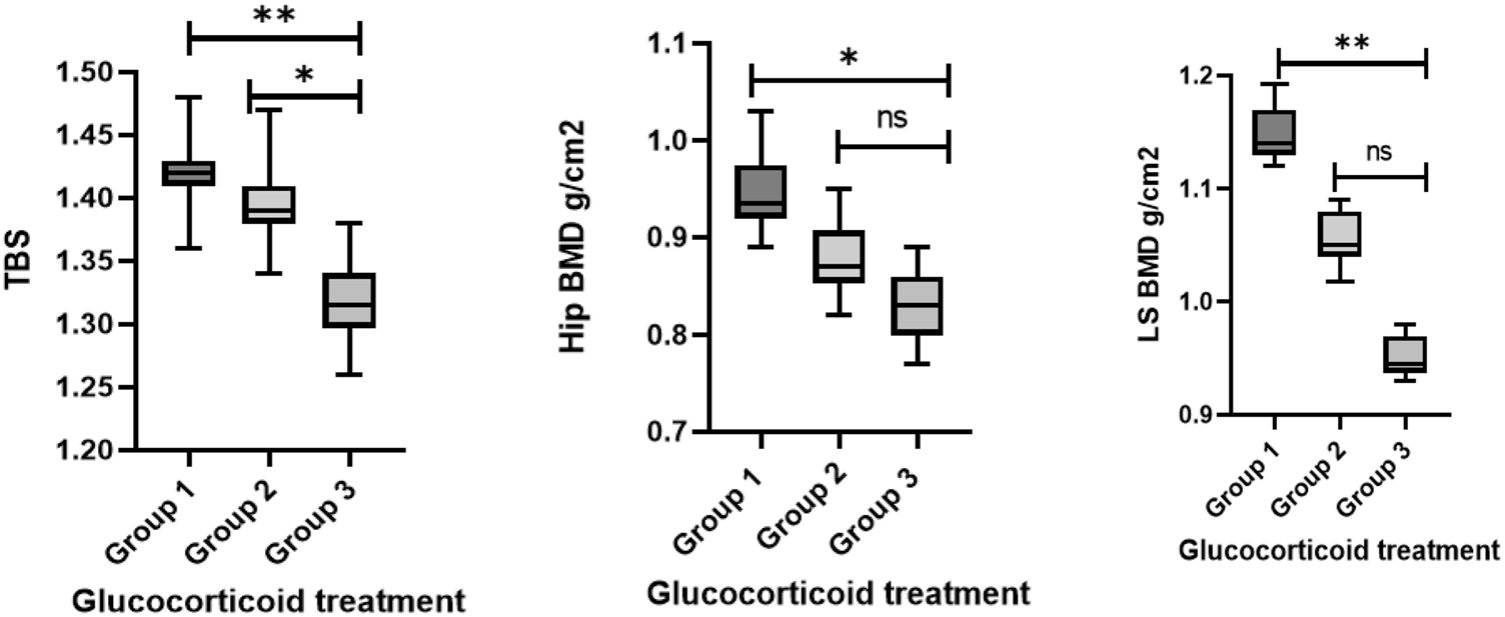
Differences between DXA parameters in the groups 1, 2, 3 according to glucocorticoids exposure, using general linear model adjusting for age, gender, disease duration, age at diagnosis. *p < 0.05, **p < 0.001, ns no significance.

Analysis of previous exposure to high-dose glucocorticoids between the study groups, after adjusting for disease duration, age, age of diagnosis and gender, in general linear model analysis revealed that the LS BMD (1.14 ± 0.01 vs. 0.95 ± 0.01 g/cm^2^, *p* = 0.001), hip BMD (0.95 ± 0.03 vs. 0.82 ± 0.03 g/cm^2^, *p* = 0.02) and TBS (1.42 ± 0.02 vs. 1.31 ± 0.03, *p* = 0.005) values in group 1 were higher than those in group 3. However, only the TBS was statistically decreased in group 3 as compared with group 2 (1.31 ± 0.03 vs. 1.39 ± 0.02, *p* = 0.012) (Fig. 1).

#### Impact of clinical disease activity, pattern and other parameters on DXA parameters

When we analysed all IBD patients, TBS positively correlated with LS BMD (*r* = 0.565, *p* < 0.005) and hip BMD (*r* = 0.470, *p* < 0.005). We found a positive correlation between ASMI and both LS BMD (*r* = 0.401, *p* < 0.005), and hip BMD (*r* = 0.438, *p* < 0.005), but also between ASMI and TBS (*r* = 0.417, *p* < 0.005). Twenty-five hydroxy vitamin D did not correlate with TBS (*r* = 0.109, *p* = 0.33), LS BMD (*r* = 0.033, *p* = 0.7) and hip BMD (*r* = 0.109, *p* = 0.33).

Considering exclusively CD patients, ASMI continued to positively correlate with LS BMD (*r* = 0.401, *p* < 0.005) and hip BMD (*r* = 0.498, *p* < 0.005), while TBS was positively associated with ASMI (*r* = 0.472, *p* = 0.001) and LS BMD (*r* = 0.552, *p* < 0.001).

When we analysed the impact of disease activity status on bone parameters, we did not find any relationship between higher clinical disease activity and LS BMD (*r* =  − 271, *p* = 0.06) or hip BMD (*r* =  − 0.02, *p* = 0.89), either in CD nor in UC patients, but there was a significant inverse correlation between higher HBI score and TBS (*r* =  − 0.300, *p* = 0.03) in CD patients.

Compared to those in remission, clinically active CD patients had a statistically increased faecal calprotectin level 175 (295) vs. 31.5 ( 74) μg/g, *p* = 0.005) and a lower TBS value (1.34 ± 0.12 vs. 1.41 ± 0.88, *p* = 0.02) (Table 3).

**Table 3:** Differences between demographic, clinical, inflammatory and DXA parameters in CD patients.

Parameter	All (48)	Remission (28)	Clinical active disease (20)	p-value
Age (year) (IQR)	42 (23)	37 (20)	47 (30)	0.9
BMI (kg/m^2^) (IQR)	24.4 (7.3)	24.6 (6.9)	24 (7.3)	0.5
Fecal calprotectin (mcg/g) (IQR)	55 (185)	31.5 (74)	175 (295)	0.005
Lumbar BMD (g/cm^2^) SD	1.04 ± 0.18	1.06 ± 0.16	1.0 ± 0.2	0.7
Hip BMD (g/cm^2^) SD	0.87 ± 0.14	0.86 ± 0.16	0.88 ± 0.12	0.5
TBS SD	1.38 ± 0.1	1.41 ± 0.88	1.34 ± 0.12	0.02
ASMI (kg/m^2^) SD	6.87 ± 1.32	7.08 ± 0.12	6.59 ± 0.13	0.3
25 hydroxy vitamin D (ng/ml) SD	19.92 ± 7.6	20.08 ± 7.4	19.6 ± 8.3	0.8

Values are presented as mean ± SD or median (IQR), number, according to the type of variable and the normality of distribution.

p-value presented statistically significant differences p < 0.05.

*BMI *body mass index BMD bone mineral density, *TBS *trabecular bone score, *ASMI* appendicular skeletal muscular index.

Moreover, the CD patients with a stricturing/penetrating pattern, had significantly lower LS BMD (0.92 ± 0.19 vs. 1.07 ± 0.1 g/cm^2^, *p* = 0.013), TBS (1.32 ± 0.13 vs. 1.40 ± 0.9, *p* = 0.031) and ASMI (6.03 ± 1.2 vs. 7.13 ± 1.26 kg/m^2^, *p* = 0.015) values than those with a non-stricturing pattern; although they also had lower values for hip BMD (0.8 ± 0.15 vs. 0.89 ± 0.14 g/cm^2^, *p* = 0.082), the result did not reach statistical significance. The localisation of CD (colonic vs. non-colonic) and age at diagnosis did not clearly show statistical significance on any of the DXA parameters studied. Furthermore, differences in patients with UC according to the Montreal classification were not detected.

Multivariate regression analysis of TBS determinants using a model that included glucocorticoid exposure for more than four courses, ASMI, LS BMD, stricturing pattern, age and gender revealed that HBI score was an independent predictor of low TBS in the CD patients (Table 4).

**Table 4:** Determinants of TBS values in CD patients.

Dependent variable	Independent variable	Unstandardized coefficients (B)	95%CI low	95%CI high	t	p
TBS r^2^ = 0.4, p = 0.02	Gender	−0.003	−0.074	0.067	−0.09	0.9
	Pattern (structuring)	−0.04	−0.123	0.041	−1.003	0.3
	Age (years)	−0.064	−0.302	0.174	−0.551	0.5
	ASMI (kg/m^2^)	0.011	−0.02	0.043	0.741	0.4
	Glucocorticoid exposure > 4 courses	−0.03	−0.108	0.049	−0.765	0.4
	HBI	−0.094	−0.183	−0.005	−2.165	0.03
	LS BMD (g/cm^2^)	0.123	−0.092	0.339	1.171	0.2

*TBS* trabecular bone score, *ASMI* appendicular skeletal mass index, *HBI* Harvey Bradshaw Index, *LS* BMD lumbar spine bone mineral density.

## Discussions

The TBS is a relatively new measure in bone quality assessment such that few studies have evaluated its usefulness in patients with IBD. To the best of our knowledge, this is the first study to provide an in-depth description of IBD activity with regard to bone abnormalities. Research has shown that IBD patients have an increased risk of low BMD and fractures, with several factors being involved, such as high-dose long-term glucocorticoid use, chronic inflammation and malabsorption^14^.

This study found that low BMD status was more prevalent in IBD patients compared with controls, which is consistent with the results of a study that showed an increased risk of low BMD for these patients compared with the general population^15^. All DXA parameters in the IBD patients were statistically lower than those in the controls. We did not find differences in DXA parameters between CD and UC patients and similar results were also reported by Lima^16^. However, the literature regarding the subject is contradictory. Ezzat et al.^17^ and Jahnsen^18^ obtained lower values in CD patients than in UC patients; nevertheless, differences in BMI and exposure to glucocorticoids could have influenced their results.

Because glucocorticoid exposure is an important enhancer of bone loss, we divided the patients into three groups according to their previous exposure to better characterise its effect on them. As expected, multiple long-term glucocorticoid exposure led to decreased DXA parameters compared with non-exposure or a single course of therapy. Furthermore, when we compared patients with different levels of glucocorticoid exposure, after adjusting for multiple factors such as gender, disease duration, age of diagnosis, and age, the only significantly different bone parameter was TBS. This could indicate that TBS is a more sensitive parameter for detecting subtle bone abnormalities induced by glucocorticoid exposure.

We found moderately statistically significant correlations between TBS and LS BMD as well as between TBS and hip BMD, indicating that the TBS could provide complementary information for the detection of bone damage in patients. Stockbrugger et al.^19^ showed discordance between decreased BMD and the prevalence of vertebral fractures, raising the need for an additional parameter. The TBS could improve the identification of risk fracture. In a recent study, Lee et al.^20^ demonstrated that TBS is significantly associated with vertebral fractures in patients with osteopenia and controls. Moreover, low lean mass has been reported to have a correlation with BMD^21^; our study observed a similar relationship between ASMI and TBS, highlighting the role of muscle in both the quantity and quality of bone.

We also found that clinical disease activity is negatively correlated with TBS in the CD patients. CD is known to involve widespread inflammation, which could account for the differences between CD and UC. Moreover, stricturing CD was more likely to have lower TBS and LS BMD values than was non-stricturing CD, suggesting that the bone of patients with complicated CD is more commonly subject to profound inflammation. Previous studies have described the same relationship between profound inflammation and bone impairment in CD. For example, Lima et al.^9^ found that low BMD and disease severity were associated with penetrating disease in CD and osteopenia, respectively. Regarding differences between CD and UC, Haschka et al.^22^ found significantly lower cortical area and thickness in CD patients with a more severe bone phenotype using HRpQCT; they also reported that both trabecular BMD volume and thickness were diminished in CD patients. Similarly, Krajicovicova et al.^8^ observed decreased TBS and severity in CD patients, with only the TBS being correlated with stricturing CD, which could be attributed to their cohort of younger patients without glucocorticoid exposure.

Furthermore, when DXA parameters were analysed between clinical disease activity and remission in the CD patients, only the TBS significantly differed, suggesting that it serves as a predictive factor for bone loss. Moreover, clinical activity is an independent factor for TBS. The fact that most of the patients were receiving glucocorticoid treatment owing to their active disease could be a confounding variable. After adjusting this parameter and other factors that could influence the results, such as ASMI, stricturing pattern and LS BMD, age, gender, we determined HBI score to be an independent factor for lower TBS in the CD patients. This indicates that the disease itself could aggravate bone deterioration to a greater extent compared with the use of corticoids. The HBI score was chosen for clinical disease assessment because it is simple to calculate and has a strong correlation with the CDAI (Crohn's disease activity index)^23^, which is used in most clinical trials to define active illness. However, we are aware of the fact that this score considers symptoms over the last few days, but it also includes other parameters as arthralgia, new fistula, abscess, anal fissures, aphthous ulcers, erythema nodosum, pyoderma gangrenosum, which are associated with a more complex disease. One additional useful parameter could be the duration of the active disease in these patients; however, this could be studied in future prospective studies. Orlic et al.^24^ showed a similar relationship between low BMD and disease activity regardless of the cumulative dose of glucocorticoids. Boussoualim et al.^25^ evaluated this relationship in axial spondylarthritis and found that higher scores of activity were associated with lower TBS values.

One of the strengths of this study is its tertiary multicentre assessment, which clearly indicates that the study patients had severe forms of the disease with worse outcomes. By contrast, the small number of patients that the statistical tests narrowed down to (multiple linear regression and confounding factors) is a limitation of this study. Moreover, the small number of patients with fragility fractures did not allow us to evaluate whether the TBS could be a good predictive parameter. Another limitation of this study is that the patients were not taking vitamin D supplements, with their mean vitamin D levels being borderline between insufficiency and deficiency, which could have influenced the TBS results. Further prospective studies should be conducted with prospective follow-up of TBS changes in comparison with BMD changes during different stages of the disease and after vitamin D supplementation.

Our findings imply that all DXA measures are lower in IBD patients than in healthy people, and that low BMD is a common consequence, often neglected. The TBS should be incorporated in the assessment of early bone impairment in patients with IBD, especially in those receiving high-dose glucocorticoids. Moreover, the TBS represents a more sensitive parameter in characterising bone impairment in active CD patients, because a lower value thereof is associated with higher disease activity. Achieving remission for this patient population could decrease the deterioration of their bone microarchitecture. Follow-up studies are warranted to further strengthen these findings.
